# LL3, a homolog of LONESOME HIGHWAY, regulates vascular cell proliferation in the root apical meristem

**DOI:** 10.1093/pcp/pcaf121

**Published:** 2025-09-26

**Authors:** Kyoko Ohashi-Ito, Marino Mori, Kuninori Iwamoto, Hiroo Fukuda

**Affiliations:** Department of Biological Sciences, Graduate School of Science, The University of Tokyo, 7-3-1 Hongo, Bunkyo-ku, Tokyo 113-0033, Japan; Department of Biological Sciences, Graduate School of Science, The University of Tokyo, 7-3-1 Hongo, Bunkyo-ku, Tokyo 113-0033, Japan; Department of Biological Sciences, Graduate School of Science, The University of Tokyo, 7-3-1 Hongo, Bunkyo-ku, Tokyo 113-0033, Japan; Akita Prefectural University, 241-438 Kaidobata-nishi, Nakano, Shimoshinjo, Akita-shi, Akita 010-0195, Japan

**Keywords:** vascular development, LHW, root, xylem, phloem

## Abstract

Vascular bundles, composed of various cell types, are essential for the transport of water and various molecules throughout the plant body. Transcriptional complexes consisting of LONESOME HIGHWAY (LHW) and TARGET OF MONOPTEROS5 regulate vascular development, particularly in two aspects: vascular cell proliferation, which increases the number of vascular cell files, and xylem differentiation in the Arabidopsis root. LHW has three homologs: LHW-LIKE 1 (LL1), LHW-LIKE 2 (LL2), and LHW-LIKE 3 (LL3). In our previous study, we demonstrated that LL1 predominantly contributes to xylem differentiation together with LHW, while its involvement in vascular cell proliferation appears to be limited. The involvement of homologs other than LHW in vascular cell proliferation remains unknown, despite the critical importance of vascular cell proliferation in the initial process of vascular development. Therefore, we investigated the roles of LL2 and LL3 in vascular cell proliferation in this study. Although single loss-of-function mutants of *ll2* and *ll3* did not exhibit obvious phenotypes, the *lhw ll3* double mutant displayed severe defects in root vascular development. In *lhw ll3* roots, only one or a few vascular cells were formed, where phloem differentiation was observed but xylem differentiation was absent. In addition, introducing *LL3* into *lhw* could rescue the *lhw* phenotype. These results suggest that LL3 has a redundant role with LHW in root vascular cell proliferation, and that both LHW and LL3 are essential regulators for this process. Thus, our work indicates that different LHW homologs contribute to distinct functions of LHW in root vascular development.

## Introduction

Vascular bundles are essential for both conducting and supporting plant tissues. They extend throughout the plant body to transport water and molecules, and contain structural cells with thick secondary cell walls that contribute to mechanical support. A vascular bundle consists of several cell types such as xylem cells, phloem cells, and procambial/cambial cells. Thus, the increase in the number of cell files through periclinal division of vascular cells—i.e. vascular cell proliferation—is a crucial step in the initial process of vascular development. The initial process of vascular development is regulated by two families of bHLH proteins named LONESOME HIGHWAY (LHW) and TARGET OF MONOPTEROS5 (TMO5) in the Arabidopsis root (Ohashi-Ito and Bergmann 2007, De Rybel et al. 2013, Ohashi-Ito et al. 2013b). A LHW family protein and a TMO5 family protein form a heterodimer (LHW–TMO5) that functions as a transcription factor in vascular development (Ohashi-Ito and Bergmann 2007, De Rybel et al. 2013, Mor et al. 2022). Indeed, the *lhw* loss-of-function mutants show a reduction in the number of vascular cell files in the roots, and the double mutant of *tmo5* and its close homolog *tmo5-like1* also shows the very similar phenotype to the *lhw* mutant. The LHW–TMO5 heterodimer directly upregulates *LONELY GUY3 (LOG3)* and *LOG4* genes, which encode enzymes for the last step of cytokinin production in the root apical meristem (RAM) (De Rybel et al. 2014, Ohashi-Ito et al. 2014). Cytokinin production and signaling are essential for vascular cell proliferation in the RAM (Sun et al. 2023). The synthesized cytokinin under the regulation of LHW–TMO5 contributes to inducing periclinal division of vascular cells, leading to an increase in their number. Thus, LHW–TMO5 is a key regulator in vascular cell proliferation during the initial process of vascular development. Additionally, LHW–TMO5 regulates xylem differentiation in the root. In the *lhw* and *tmo5 t5l1* mutants, xylem vessel differentiation is partly suppressed in the root (Ohashi-Ito and Bergmann 2007, De Rybel et al. 2013). Also, LHW–TMO5 positively regulates master regulators for xylem vessel differentiation, *VASCULAR-RELATED NAC-DOMAIN* genes (*VND*s) (Ohashi-Ito et al. 2024). Thus, LHW–TMO5 is considered to have at least two functions: vascular cell proliferation and xylem differentiation in root vascular development.

The *LHW* gene family comprises four members: *LHW*, *LL1* (also known as *LHL3*), *LL2* (also known as *LHL2*), and *LL3* (also known as *LHL1*) (De Rybel et al. 2013, Ohashi-Ito et al. 2013a). Among them, detailed functional analysis with LHW has been conducted only on *LL1*, revealing that LL1 shares a redundant role with LHW in a subset of its functions. The *lhw ll1* exhibits an enhanced xylem differentiation defect compared to the *lhw* single mutant. While previous studies have reported a reduction in the number of vascular cells in the roots of both the *ll1* single mutant and the *lhw ll1* double mutant (De Rybel et al. 2013, Mor et al. 2022), our earlier research demonstrated that the vascular cell number in *lhw ll1* roots is comparable to that observed in the *lhw* single mutant (Ohashi-Ito et al. 2013a). These findings suggest that, although LL1 functions redundantly with LHW, its contribution to the regulation of vascular cell proliferation may be limited. Instead, LL1 is likely to act cooperatively with LHW more specifically in the regulation of xylem differentiation.

As mentioned above, vascular cell proliferation, which increases the number of vascular cell files, is a critical step in the initial process of vascular development. Therefore, it is essential to investigate whether other LHW homologs regulate this process to elucidate the molecular mechanism underlying vascular development. Notably, the *lhw* single mutant retains approximately half the number of vascular cells compared to the wild type, suggesting that other LHW homologs, such as LL2 and LL3, may also function redundantly in this process. The expression patterns of *LHW* and its homologs significantly overlap in vascular cells (De Rybel et al. 2013, Ohashi-Ito et al. 2013a, Mor et al. 2022), indicating that LL2 and LL3 may share redundant roles with LHW in the control of vascular cell proliferation. Consequently, we conducted a functional analysis of LL2 and LL3 in conjunction with LHW, with a particular focus on the roots, as a model of the initial process of vascular development in this study.

## Results

### Comparison of expression patterns of *LHW* and its homologs in the RAM

**Figure 1:**
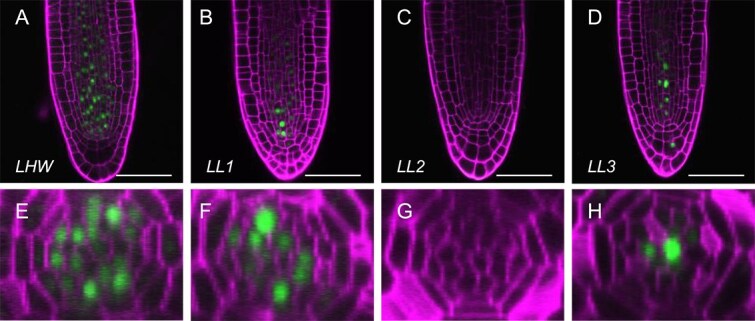
Expression patterns of *LHW*, *LL1*, *LL2*, and *LL3*. (A–D) Images of YFP-NLS driven by *LHW* (A), *LL1* (B), *LL2* (C), and *LL3* (D) promoters, respectively, in the apical meristem of lateral roots. Bars, 50 μm. (E–H) Cross-sectional view images of (A–D), respectively.

To infer which candidate homologs function together with LHW in the process of vascular cell proliferation, we compared the expression patterns of *LHW* and its homologs in the RAM of lateral roots. Consistent with previous reports, *LHW* and *LL1* were broadly expressed in the vascular cells of the meristematic region (Fig. 1A, B, E, and  F). In contrast, *LL2* expression was not observed in the RAM (Fig. 1C and G). *LL3* was preferentially expressed in xylem precursor cells within the meristematic region (Fig. 1D and H). Because a previous report showed that LL1 appears to play a limited role during vascular cell proliferation of the RAM, these results suggest that *LL3* is a likely candidate functioning in the vascular cell proliferation process.

### Loss-of-function *ll2* and *ll3* enhances the *lhw* phenotype in the root

**Figure 2:**
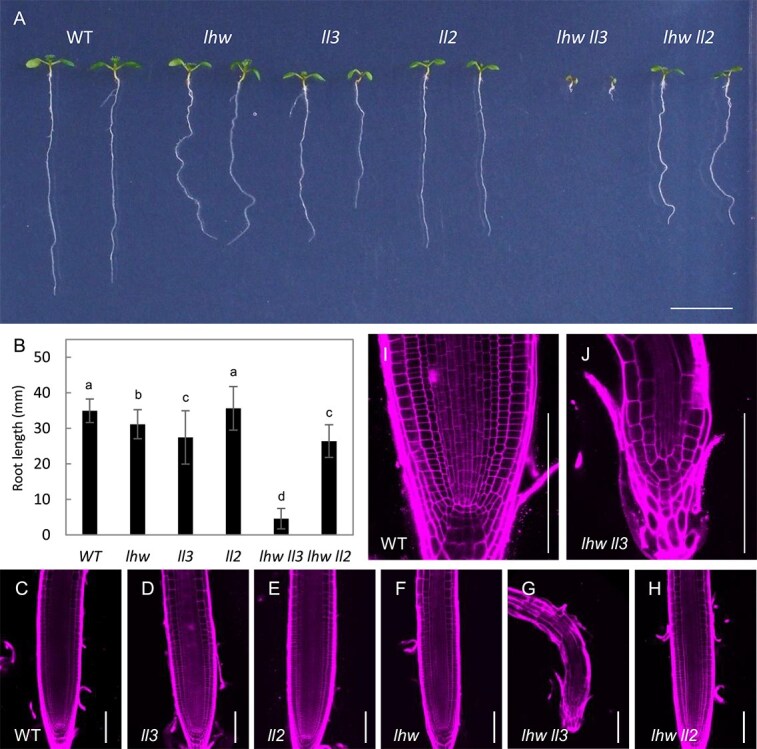
Phenotype of *lhw* homolog mutants*.* (A) An image of 7-day-old wild type, *lhw*, *ll3*, *ll2*, *lhw ll3*, and *lhw ll2* plants. Bar, 1 cm. (B) Root length of 7-day-old wild type, *lhw*, *ll3*, *ll2*, *lhw ll3*, and *lhw ll2* plants. Error bars indicate SD (*N* ≥ 24). Different letter superscripts in each column indicate significant differences (*P* < 0.01; one-way ANOVA with the Tukey–Kramer post hoc test). (C–J) Images of the root apical meristem of 7-day-old wild type (C and I), *lhw* (D), *ll3* (E), *ll2* (F), *lhw ll3* (G and J), and *lhw ll2* (H) plants. Bars, 100 μm.

To investigate whether LL2 and LL3 have functions in root vascular development, we prepared *ll2* and *ll3* loss-of-function single mutants. The *ll2* mutant was produced using a CRISPR-Cas9 system, resulting in a nucleotide insertion at the end of the first exon of *LL2*, which caused a frame shift and produced a stop codon after four amino acids from the mutation site (Supplementary Fig. S1). The *ll3* mutant included a T-DNA insertion in the ninth exon of *LL3* (Supplementary Fig. S1). We observed 7-day-old seedlings of *ll2* and *ll3* single mutants, and they showed no obvious macroscopic morphological changes (Fig. 2A). Next, we produced double mutants of *ll2* and *ll3* with *lhw*. While the seedlings of *lhw ll2* double mutants were similar to *lhw*, the *lhw ll3* double mutants showed drastic growth arrest (Fig. 2A). The *lhw ll3* seedlings were extremely small, with short roots. We then measured the root length of 7-day-old seedlings of each genotype (Fig. 2B and Supplementary Table S1). Although the length of *ll2* single mutant roots was not different from the wild type, the *lhw ll2* double mutant had slightly shorter roots than the *lhw* single mutant. On the other hand, the *ll3* single mutant had shorter roots than the wild type, which is similar to the *lhw* mutant, and the *lhw ll3* double mutant had extremely shorter roots than any other genotypes. The root tip of *lhw ll3* was also smaller to those of other genotypes (Fig. 2C–H). Observation of RAM indicated that the RAM structures of wild type, *lhw*, *ll3*, *ll2*, and *lhw ll2* were normal. However, the *lhw ll3* RAM was almost lost, in which the quiescent center and meristematic cells were absent (Fig. 2I and J). These results suggest that LL3 plays a critical role in the regulation of root development together with LHW.

### L‌L3 and LHW functions are crucial for vascular cell proliferation in the root

**Figure 3:**
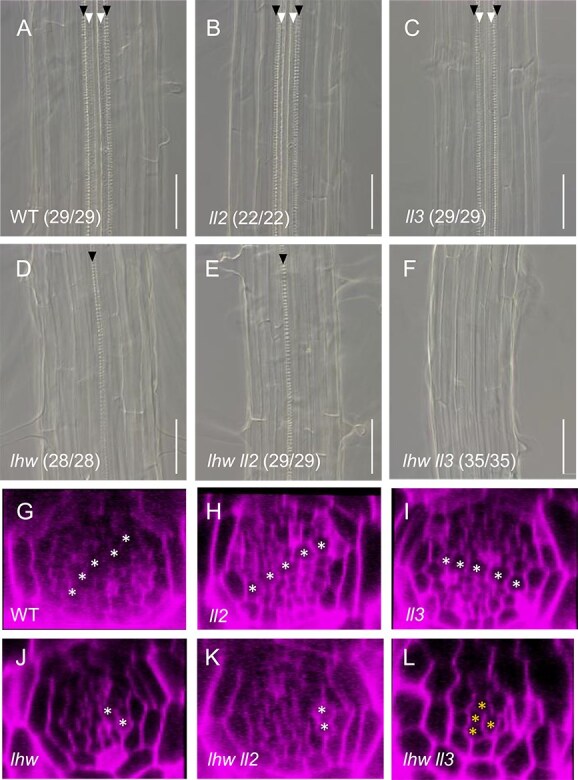
Vascular phenotype of *lhw*, *ll2*, and *ll3* mutant roots. (A–F) DIC images of 7-day-old wild type (A), *ll2* (B), *ll3* (C), *lhw* (D), *lhw ll2* (E), and *lhw ll3* (F) differentiation zone of roots. Filled and open triangles indicate a protoxylem and a metaxylem vessel. The fraction of samples showing similar patterns is presented. Bars, 50 μm. (G–L) Cross-sectional views of the vascular tissue of root in 7-day-old wild type (G), *ll2* (H), *ll3* (I), *lhw* (J), *lhw ll2* (K), and *lhw ll3* (L). Asterisks indicate xylem cells (G-K). and vascular cells (L).

Next, we investigated root vascular phenotypes of these mutants in detail. Xylem differentiation in the *ll2* and *ll3* roots was the same as in the wild type, which includes two protoxylem vessels and two to three metaxylem vessels (Fig. 3A–C). In the *lhw ll2* root, only one protoxylem vessel was observed, which is similar to the *lhw* root (Fig. 3D and E). In the *lhw ll3* roots, however, no differentiated xylem vessel was observed (Fig. 3F). We then examined the root vascular pattern of each genotype (Fig. 3G–L). The vascular pattern of wild type, *ll2* and *ll3* roots was a typical diarch pattern, in which a xylem axis exists in a line and procambial/phloem cells located on either side of the xylem axis (Fig. 3G–I). The *lhw* and *lhw ll2* roots had a monarch pattern, with only one protoxylem and one phloem region (Fig. 3J and K). The *lhw ll3* root had only a few vascular cells without a clear pattern (Fig. 3L). In summary, both *ll2* and *ll3* single mutants exhibited phenotypes almost identical to the wild type. However, *ll3*, not *ll2*, enhances *lhw* phenotypes in the root vascular tissue. The considerable reduction of the number of vascular cells in the *lhw ll3* suggests that LL3 has a role in regulating vascular cell proliferation. Therefore, we decided to focus on LL3 for subsequent experiments.

**Figure 4:**
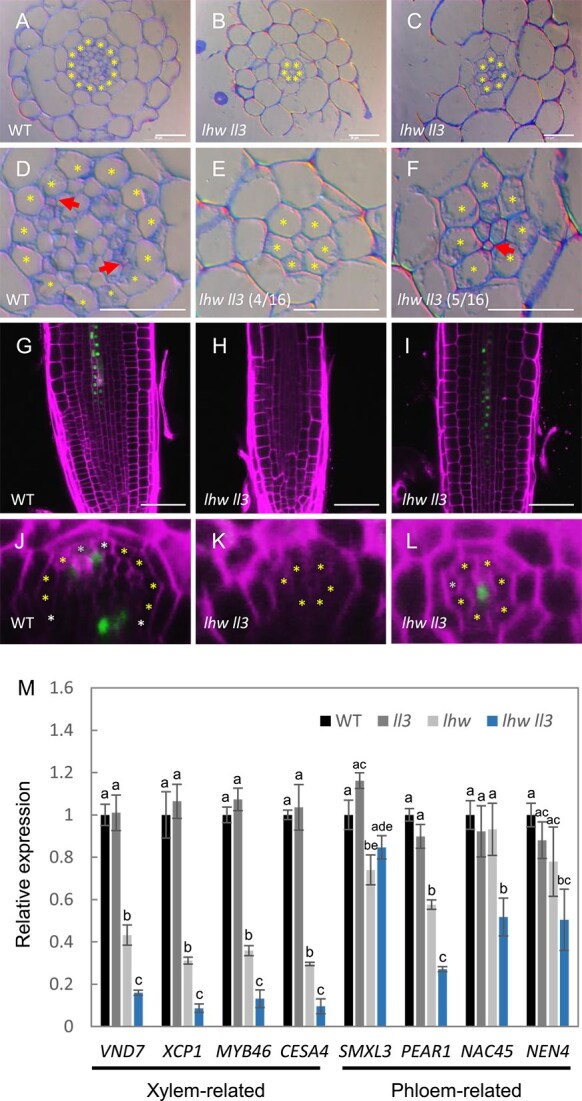
Vascular phenotype of the *lhw ll3* root. (A–F) Cross-sections of the roots of 7-day-old wild type (A and D) and *lhw ll3* (B, C, E, and F). (D–F) are enlarged views of vascular region in (A–C), respectively. Asterisks indicate pericycle cells. Arrows indicate sieve elements. The fraction of samples showing similar patterns is presented. Bars, 20 μm. (G–I) Images of *APL promoter::YFP-NLS* in 7-day-old root of wild type (G) and *lhw ll3* (H and I). Bars, 50 μm. (J–L) Cross view images of *APL promoter::YFP-NLS* in 7-day-old root of wild type (J) and *lhw ll3* (K and L). Asterisks indicate pericycle cells. (M) Quantitative RT-PCR analysis of expression levels of xylem and phloem differentiation-related genes in 7-day-old wild type, *ll3*, *lhw*, and *lhw ll3* roots. Bars from left to right represent wild type, *ll3*, *lhw*, and *lhw ll3*. The expression level of each gene in wild type was normalized to 1.0. Data represent mean ± SD. Different letter superscripts in each column indicate significant differences (*P* < .001; one-way ANOVA with the Tukey–Kramer post hoc test). The mean is average of three independent biological replicates.

To examine the vascular tissue of *lhw ll3* in more detail, we performed cross-sections of *lhw ll3* roots (Fig. 4). In the *lhw ll3* roots, the number of vascular cells was severely decreased compared to that in the wild type. In *lhw ll3* roots, the number of vascular cells ranged from as few as 1 to as many as 6, with an average of 2.8 (*n* = 16) in our growth condition. In the *lhw ll3* root with only one vascular cell, it did not differentiate into a vessel or sieve element (4/16, Fig. 4B and E). However, in the *lhw ll3* root with a few vascular cells, a sieve element-like cell, but not a vessel, was observed (5/16, Fig. 4C and F). To clarify whether vascular cells in the *lhw ll3* root differentiate into phloem cells, we introduced a phloem marker, *APL promoter YFP-NLS* into the *lhw ll3* plants (Bonke et al. 2003). The signal of *APL promoter YFP-NLS* was observed in two phloem regions in the wild type root (Fig. 4G and J). In the vascular cells of the *lhw ll3* roots, the signal was absent or restricted to a single region (Fig. 4H, I, K, and  L). The *lhw ll3* roots without the signal were thinner and had fewer vascular cells than the roots with the signal. We further examined the expression levels of representative genes involved in xylem vessel differentiation (e.g. *VND7*, *XCP1, MYB46*, and *CESA4*) and phloem/sieve element differentiation (e.g. *SMXL3, PEAR1*, *NAC45*, and *NEN4*) in the *ll3*, *lhw*, and *lhw ll3* roots (Kubo et al. 2005, Ohashi-Ito et al. 2010, Furuta et al. 2014, Wallner et al. 2017, Miyashima et al. 2019). In the *ll3* roots, the expression levels of all examined genes were comparable to those in wild-type roots (Fig. 4M, Supplementary Table S2). In the *lhw* roots, however, the expression of genes related to xylem vessel differentiation was reduced by ~60%, whereas the expression of phloem-related genes remained unchanged or showed a moderate decrease. In the *lhw ll3* roots, the expression of xylem vessel differentiation-related genes was markedly reduced, whereas the reduction of phloem differentiation-related genes was relatively moderate. These results suggest that vascular cells in the *lhw ll3* root fail to differentiate into xylem vessels, but in some cases, they differentiate into sieve elements depending on the number of vascular cells.

### L‌L3 interacts with TMO5, T5L1, and SACL proteins

Previous studies have shown that the LHW protein forms heterodimers with members of the TMO5 and SACL families, but does not form homodimers with itself (Ohashi-Ito and Bergmann 2007, De Rybel et al. 2013, Katayama et al. 2015, Vera-Sirera et al. 2015). To characterize the LL3 protein, we examined its interactions with these bHLH proteins using a Bimolecular Fluorescence Complementation assay (BiFC). BiFC signals were detected between LL3 and TMO5, T5L1, or SACL3, but not between LL3 and SACL2 or LL3 itself, indicating that LL3 forms heterodimers at least with TMO5, T5L1, and SACL3 (Supplementary Fig. S2). These results suggest that LL3 shares fundamental functional characteristics with the LHW protein.

### L‌L3 redundantly regulates the targets of LHW

**Figure 5:**
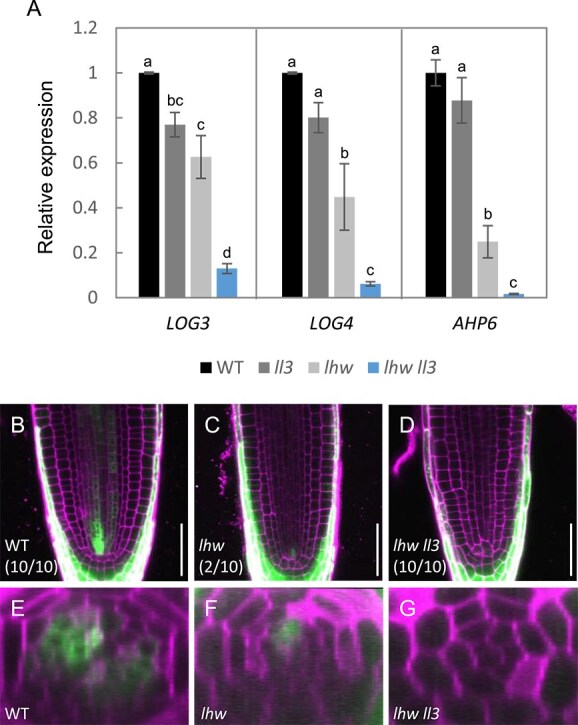
Expression of targets of LHW. (A) Quantitative RT-PCR analysis of *LOG3, LOG4*, and *AHP6* expression levels in 7-day-old wild type, *ll3*, *lhw*, and *lhw ll3* roots. Bars from left to right represent wild type, *ll3*, *lhw*, and *lhw ll3*. The expression level of each gene in wild type was normalized to 1.0. Data represent mean ± SD. Different letter superscripts in each column indicate significant differences (*P* < 0.001; one-way ANOVA with the Tukey–Kramer post hoc test). The mean is average of three independent biological replicates. (B–D) Expression patterns of TCSn::GFP in the RAM of 7-day-old wild type (B), *lhw* (C), and *lhw ll3* (D). The fraction of samples showing similar patterns is presented. Bars, 50 μm. (E–G) Cross-sectional views of the vascular region shown in panels (B–D).

To further evaluate the redundant role of LL3 with LHW in root vascular development, we compared the expression levels of direct target genes of LHW such as *LOG3*, *LOG4*, and *AHP6* (De Rybel et al. 2013, Ohashi-Ito et al. 2014). The expression level of *LOG3* was reduced in the *ll3* mutant, further reduced in the *lhw* mutant, and most significantly reduced in the *lhw ll3* mutant. Similarly, the expression levels of *LOG4* and *AHP6* showed a decreasing trend in the *ll3* mutant, although these changes were not statistically significant. These genes were expressed at lower levels in the *lhw* mutant, and substantially reduced in the *lhw ll3* mutant (Fig. 5A). This result is consistent with the idea that LL3 and LHW redundantly regulate the gene expression in the root vascular development. Since previous studies have shown that LHW together with TMO5 regulates vascular cell proliferation through cytokinin response in the vascular region of the RAM, we next examined cytokinin response using the marker *TCSn-GFP* in the *lhw* and *lhw ll3* RAM (Zürcher et al. 2013). The signal of *TCSn-GFP* was observed in two regions of procambium/phloem cells in the wild-type vascular tissue in the RAM (Fig. 5B and E). In the vascular region of the *lhw* RAM, signal was detected in 2 out of 10 plants, while the remaining 8 showed no signal (Fig. 5C and F). In contrast, signal in the vascular region of RAM was not observed in any of the 10 plants of the *lhw ll3* (Fig. 5D and G). These results also support the notion that LL3 has a redundant role with LHW in the regulation of the process of vascular cell proliferation through cytokinin response.

**Figure 6:**
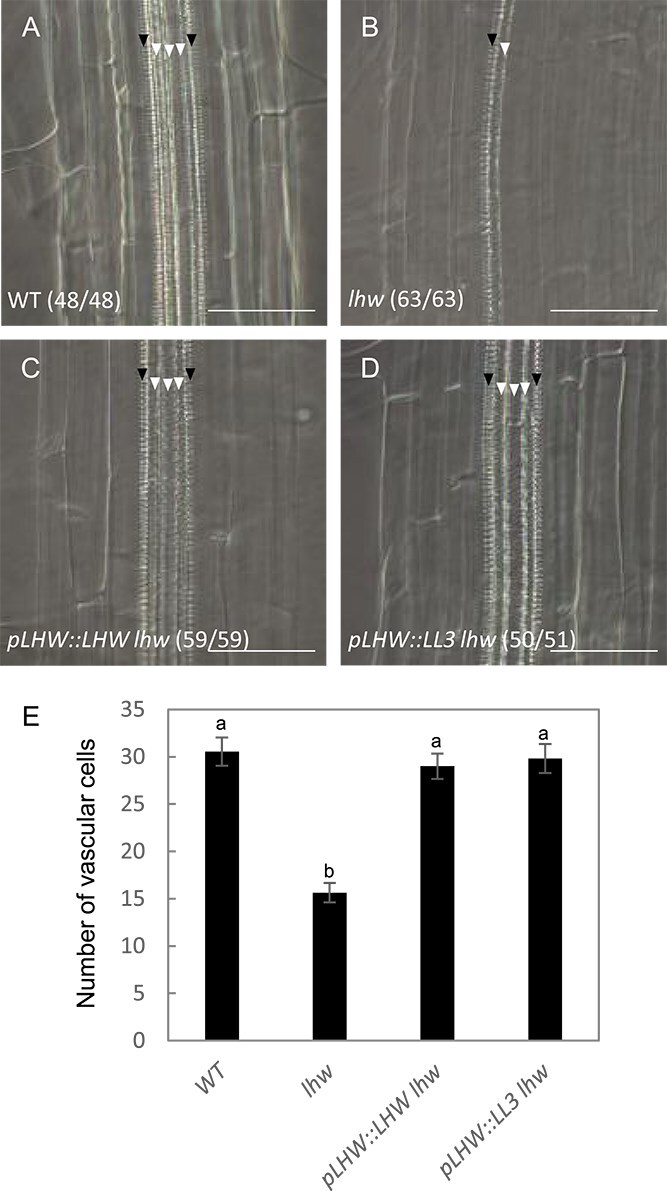
Complementation analysis of *LL3* in *lhw*. (A–D) DIC images of 7-day-old wild type (A), *lhw* (B), *pLHW::LHW lhw* (C), and *pLHW::LL3 lhw* (D) differentiation zone of roots. Filled and open triangles indicate a protoxylem and a metaxylem vessel. The fraction of samples showing similar patterns is presented. Bars, 50 μm. E: Numbers of vascular cells in wild type, *lhw*, *pLHW::LHW lhw*, and *pLHW::LL3 lhw* roots. Data represent mean ± SD. Different letter superscripts in each column indicate significant differences (*P* < 0.01; one-way ANOVA with the Tukey–Kramer post hoc test).

Finally, to further confirm the LL3 function, we performed complementation tests, in which *LL3* driven by the *LHW* promoter was introduced into the *lhw* mutant (*pLHW::LL3 lhw*). The monarch vascular pattern of the *lhw* root is the most obvious readout of the *lhw* phenotype (Ohashi-Ito and Bergmann 2007). The vascular patterns of *pLHW::LL3 lhw* root showed the diarch pattern like those of wild type and *pLHW::LHW lhw* roots (Fig. 6A–D). Introduction of *pLHW::LL3* in the *lhw* mutant also restored the number of vascular cells to near wild-type levels (Fig. 6E and Supplementary Table S3). Thus, LL3 complemented the *lhw* root phenotype. These results suggest that LL3 has a function almost identical to LHW in the root vascular development.

## Discussion

In this study, we demonstrated that LL3, a homolog of LHW, plays an essential role alongside LHW in the initial process of vascular development in the root. The loss of LL3 and LHW functions caused a significant decrease in the number of vascular cells in the root. In the most severe phenotype of the *lhw ll3* root, only one vascular cell was formed, and no sign of periclinal division was observed. This result suggests that LL3 and LHW are indispensable factors for vascular cell proliferation, increasing the number of vascular cell files in the root. Our qRT-PCR analysis indicated that the target genes of LHW were also regulated by LL3, and complementation analysis demonstrated that LL3 can substitute for the function of LHW. These results further suggest that LL3 has a redundant role with LHW in the regulation of vascular cell proliferation at the initial step of vascular development. Because the obvious phenotype in root vascular development was observed only in the *lhw* single mutant but not in the *ll3* mutant, LL3 likely plays a secondary role compared to LHW in the initial process of root vascular development. Previous studies have shown that the expression of *LHW* and its homologs is partly overlapping and partly different in various organs and meristems, with their own expression patterns (De Rybel et al. 2013, Ohashi-Ito et al. 2013a, Mor et al. 2022). We previously reported that *LL3* expression signals were not detected in torpedo, linear, or mature embryos by *in situ* hybridization (Ohashi-Ito et al. 2013a). However, *LL3* expression at other embryonic stages remains unknown. In contrast, *LHW* expression begins before the stage at which vascular cells are formed and continues thereafter. The apparent difference in timing of expression between *LHW* and *LL3* during embryogenesis may account for the observed phenotypic difference between *lhw* and *ll3* mutants. In addition, in this study, we demonstrated that *LL3* is expressed in xylem precursor cells in the RAM. This expression pattern overlaps with that of *LHW*, although *LHW* expression in xylem precursor cells was relatively weaker compared to other vascular cells. We have indicated that xylem precursor cells act as a signal center of proliferation of vascular cells in the RAM (Ohashi-Ito et al. 2014). The expression pattern of *LL3* is consistent with its proposed role in promoting vascular cell proliferation. The expression patterns of *LL3* and *LHW* appear to complement each other to some extent. Thus, the functional differences between LHW and LL3 may be attributed to variations in their spatial and temporal expression patterns, as well as in their expression levels. The loss of *LL2* in the *lhw* enhanced the *lhw* phenotype in terms of root length, but not of the vascular pattern. This observation is consistent with the undetectable level of *LL2* expression in the RAM. In contrast, a previous study reported weak expression of *LL2* in the RAM (Mor et al. 2022). This discrepancy may be attributed to the difference in promoter length and reporter type, as well as the inherently low expression levels of *LL2*. It has also been reported that the root of the single *ll2* mutant has a slightly reduced number of vascular cells (Mor et al. 2022). Therefore, we cannot exclude the possibility that LL2 may also act at vascular cell proliferation in the RAM. Our previous study showed that LL1 preferentially plays a redundant role with LHW in the process of xylem differentiation (Ohashi-Ito et al. 2013a). Together with these findings, this study suggests that the LHW family members play different roles in the RAM: LL1 mainly regulates xylem differentiation, while LL3 primarily regulates vascular cell proliferation.

We observed variation in the phenotype of *lhw ll3* roots, ranging from weak ones with a few vascular cells to strong ones with only one vascular cell. Detailed analysis with markers demonstrated that such vascular cells contain phloem cells but no xylem vessels,. This fact suggests that the establishment of phloem identity may be independent of the LHW function. In addition to enhancement of the periclinal division of vascular cells, the LHW–TMO5 heterodimer regulates xylem vessel differentiation in cell-autonomous fashion through the upregulation of *VND1*, *VND2*, and *VND3*, which are master regulators of vessel differentiation (Ohashi-Ito et al. 2024). Thus, xylem and phloem identities are likely assigned to vascular cells separately. Because phloem differentiation was observed only in the *lhw ll3* roots with few vascular cells, but not in those with just one vascular cell, cell division may be required for gaining phloem identity.

In summary, this study revealed that LL3 is an essential member of the LHW subfamily for vascular cell proliferation, and functions in producing a number of vascular cells at the initial stages of root vascular development. Together with the fact that LL1 functions in xylem differentiation, each LHW family member plays a distinct role within LHW functions.

## Materials and Methods

### Plant materials and growth conditions

Arabidopsis accession Columbia-0 (Col-0) and mutants *lhw* (SALK_079402), *ll2*, and *ll3* (CS317584) were used in this study. The *ll2* mutant was generated in Col-0 and *lhw* background using the CRISPR/Cas9 system-based pDe-CAS9 vector (Fauser et al. 2014). The *lhw ll3* double mutant was generated by crossing *lhw* and *ll3* single mutants. Transgenic lines carrying *pLHW::LHW* and *pLHW::LL3* in the *lhw* background were obtained by *Agrobacterium*-mediated transformation with the floral dipping method using the vectors described below. Seeds were sown on half-strength Murashige and Skoog (0.5× MS) agar plates containing 1% sucrose and appropriate antibiotics, incubated at 4°C for 2–3 days, and then moved to an incubator for growth under continuous light at 22°C.

### DNA manipulation

To generate the *ll2* mutant using the CRISPR/Cas9 technology, CRISPR RNA (crRNA) was designed by the CRISPRdirect web tool (Naito et al. 2015) to target and mutate the *LL2* gene. A double-stranded DNA fragment was produced by annealing LL2-CRISPR-F and LL2-CRISPR-R oligonucleotides containing the crRNA sequences. This fragment was then ligated to the *Bbs*I-digested pEN-Chimera vector (Fauser et al. 2014) using the Ligation high Ver.2 (Toyobo, Osaka, Japan), and subcloned into the pDe-CAS9 vector (Fauser et al. 2014) using LR Clonase II Enzyme Mix (Thermo Fisher Scientific, Waltham, USA). The promoter sequence of *LHW* (−5000 to −538), *LL1* (−4650 to −1), *LL2* (−5000 to −249), *LL3* (−1690 to −286), and *APL* (−2900 to −1) was amplified by PCR and cloned into the pENTR/D-TOPO cloning vector (Thermo Fisher Scientific), and then integrated into the pBGYN vector (Kubo et al. 2005) using LR Clonase II Enzyme Mix. The coding sequences of *LHW* and *LL3* were amplified by PCR and cloned into the pENTR/D-TOPO cloning vector. The promoter sequence of *LHW* was amplified by PCR with pLHW-NotI-F and pLHW-NotI-R primers, digested with *Not*I, and ligated to the *Not*I-digested entry vectors containing the coding sequences of *LHW* or *LL3*. The resultant plasmids were recombined with the pGWB1 vector (Nakagawa et al. 2007) using the LR Clonase II Enzyme Mix. Full-length coding sequences of *LL3*, *SACL2*, *SACL3*, *T5L1*, and *TMO5* (with or without a stop codon) amplified by PCR were cloned into the pENTR/D-TOPO cloning vector and recombined with the destination vectors pCX-GW and pXN-GW (Kim et al., 2009), which were gifts from Dr. Wolf Frommer (Addgene plasmid #48257 and #48258). A cytokinin sensor, *TCSn::GFP*, in the pCB302 backbone was a gift from Bruno Müller (Zürcher et al. 2013). Oligonucleotides used in this study are listed in Supplementary Table S4.

### Total RNA isolation and quantitative RT-PCR

Total RNA isolation and cDNA synthesis were performed as described previously (Ohashi-Ito et al. 2010). Quantitative RT-PCR was conducted using TaqPRO Universal SYBR qPCR Master Mix (Vazyme, Nanjing, China) on LightCycler 480 Instrument II (Roche, Basel, Switzerland), according to the manufacturer’s instructions. Standard curves for each primer set were created by the second derivative maximum method using 10-fold serial dilutions of cDNA obtained from one of the samples. Expression levels of each gene were calculated from the standard curves, and relative expression levels were determined using *UBQ10* as the reference gene. Oligonucleotides used for the qRT-PCR analysis are listed in Supplementary Table S4.

### Histological analysis and fluorescence imaging

Fluorescence imaging was performed using an FV1200 confocal microscope (Olympus, Tokyo, Japan) and a BX51 microscope (Olympus). Differential interference contrast images were acquired using a BM5500 microscope (Leica Microsystems, Wetzlar, Germany) after specimens were treated with chloral hydrate. Images of 7-day-old plants on plates were captured using a digital camera OM-D (Olympus), and root lengths were quantified using NIH ImageJ software. Cross-sections of roots were prepared using the method previously described (Ohashi-Ito et al. 2023). To count numbers of vascular cells, 4-day-old roots were fixed in 4% paraformaldehyde in phosphate-buffered saline containing 1% dimethyl sulfoxide, 0.1% TritonX-100, and 0.2% SR2200 (Renaissance Chemicals) for 30 min. Roots were observed under a FV1200 microscope (Olympus). Numbers of vascular cells were quantified at the end of meristematic zone of RAM.

### BiFC assay

Coding sequences of LL3, SACL2, SACL3, TMO5, and T5L1 were fused downstream to the C-terminal fragment of GFP in the vector pCX-GW, while coding sequences of LHW and LL3 were fused upstream to the N-terminal fragment of GFP in the vector pXN-GW. The chimeric genes were co-infiltrated into the leaves of *Nicotiana benthamiana* using agrobacterium (Ohashi-Ito and Bergmann 2006) and expressed under the control of the cauliflower mosaic virus 35S promoter for 2 days.

### AGI code

LHW; AT2G27230, LL1/LHL3; AT1G64625, LL2/LHL2; AT2G31280, LL3/LHL1; AT1G06150, TMO5; AT3G25710, T5L1; AT1G68810, SACL2; AT5G50010, SACL3; AT1G29950.

## Supplementary Material

Supplementary_Figure_S1_pcaf121

Supplementary_Figure_S2_pcaf121

pcp-2025-e-00140-File002_pcaf121

## Data Availability

The data underlying this article are available in the article and in its online supplementary material.
