# Supplementary material for: LL3, a homolog of LONESOME HIGHWAY, regulates vascular cell proliferation in the root apical meristem
Source: Plant Cell Physiol. 2025 Sep 26;67(1):20–9. doi: 10.1093/pcp/pcaf121 (PMC12814876; doi:10.1093/pcp/pcaf121)
Supplement: Supplementary_Figure_S1_pcaf121 [file supplementary_figure_s1_pcaf121.pdf]

Supplemental figure 1

A

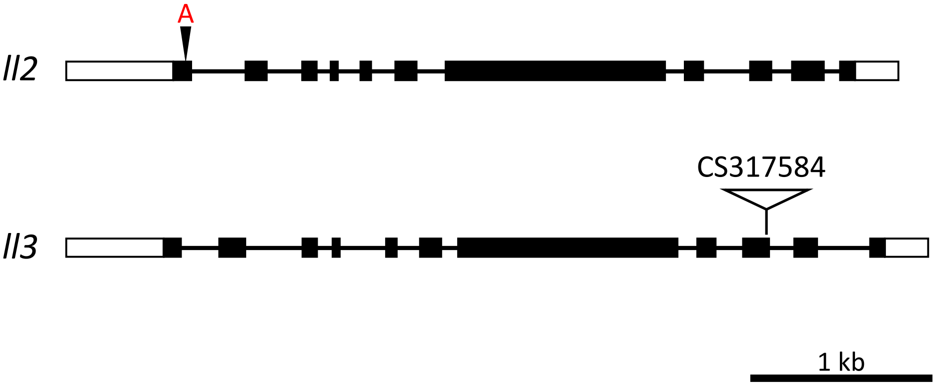

B

```
          10        20        30        40        50
WT :  ATGGGTTCTACTTCTCAAGAGATACTGAAGAGCTTTTGCTTTAACACGGA
      M  G  S  T  S  Q  E  I  L  K  S  F  C  F  N  T  D

ll2 : ATGGGTTCTACTTCTCAAGAGATACTGAAGAGCTTTTGCTTTAACACGGA
      M  G  S  T  S  Q  E  I  L  K  S  F  C  F  N  T  D

          60        70        80        90        100
WT :  CTGGGACTATGCTGTGTTCTGGCAACTTAATCATCGAGGATCTCGAATG
       W  D  Y  A  V  F  W  Q  L  N  H  R  G  S  R  M

ll2 : CTGGGACTATGCTGTGATTCTGGCAACTTAA
       W  D  Y  A  V  I  L  A  T  *
```

Supplemental figure 1 Mutations in *LL2* and *LL3*
A: Gene structures and mutation positions of *LL2* and *LL3*. White and black boxes indicate UTRs and exons, respectively. Arrowheads indicate the position of the mutation of *ll2*. The T-DNA insertion position is indicated in *LL3*.
B: Changes of nucleotides and amino acids sequences in the *ll2* mutant. The red letter “A” is inserted nucleotide in the *ll2* mutant.
