# Supplementary material for: LL3, a homolog of LONESOME HIGHWAY, regulates vascular cell proliferation in the root apical meristem
Source: Plant Cell Physiol. 2025 Sep 26;67(1):20–9. doi: 10.1093/pcp/pcaf121 (PMC12814876; doi:10.1093/pcp/pcaf121)
Supplement: Supplementary_Figure_S2_pcaf121 [file supplementary_figure_s2_pcaf121.pdf]

Supplemental figure 2

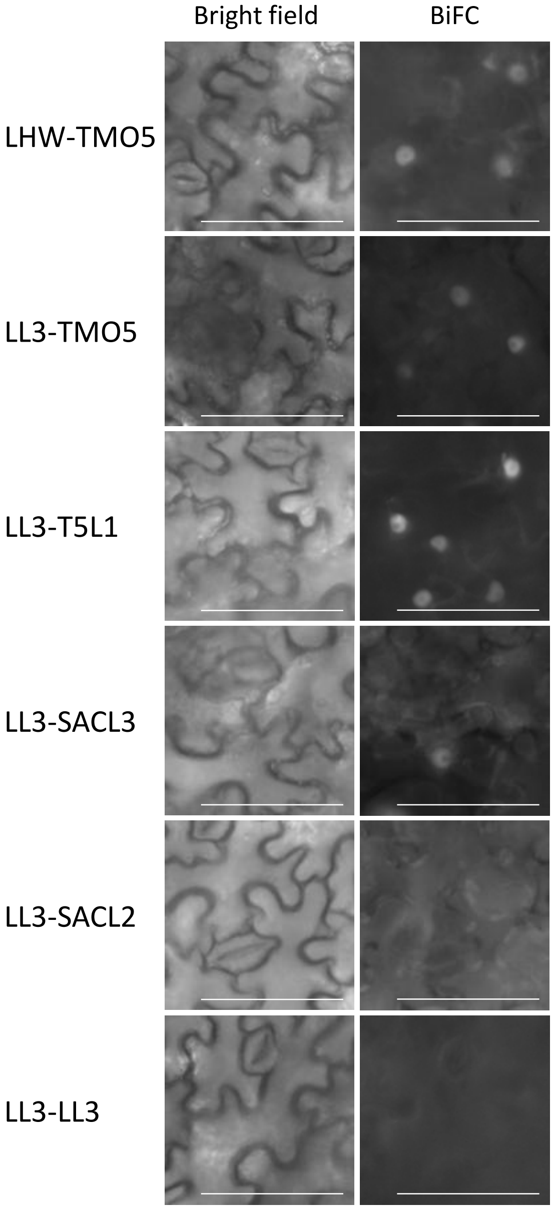

Supplemental figure 2 BiFC assay between LL3 and LHW interacting proteins
Left panels: bright-field images, right panels: GFP fluorescence images. Bars, 100 μm.
